# Antimicrobial Peptides with Anti-*Candida* Activity

**DOI:** 10.3390/ijms23169264

**Published:** 2022-08-17

**Authors:** Aitzol Perez-Rodriguez, Elena Eraso, Guillermo Quindós, Estibaliz Mateo

**Affiliations:** Department of Immunology, Microbiology and Parasitology, Faculty of Medicine and Nursing, University of the Basque Country (UPV/EHU), 48940 Leioa, Spain

**Keywords:** fungal infections, candidiasis, microbial resistance, antifungal peptides

## Abstract

Mycoses are accountable for millions of infections yearly worldwide. Invasive candidiasis is the most usual, presenting a high morbidity and mortality. *Candida albicans* remains the prevalent etiologic agent, but the incidence of other species such as *Candida parapsilosis*, *Candida glabrata* and *Candida auris* keeps increasing. These pathogens frequently show a reduced susceptibility to commonly used antifungal drugs, including polyenes, triazoles and echinocandins, and the incidence of emerging multi-drug-resistant strains of these species continues to increase. Therefore, the need to search for new molecules that target these pathogenic species in a different manner is now more urgent than ever. Nature is an almost endless source of interesting new molecules that could meet this need. Among these molecules, antimicrobial peptides, present in different sources in nature, possess some advantages over conventional antifungal agents, even with their own drawbacks, and are considered as a promising pharmacological option against a wide range of microbial infections. In this review, we describe 20 antimicrobial peptides from different origins that possess an activity against *Candida*.

## 1. Introduction

Mycoses cause millions of infections yearly [1]. Invasive candidiasis is the most usual, with a high morbidity and mortality, mainly in persons with a weakened immune system such as patients receiving chemotherapy or recovering from organ transplantation [2]. *Candida albicans* remains the most prevalent etiologic agent, but the etiological importance of other species, such as *Candida parapsilosis*, *Candida glabrata* or *Candida auris*, is increasing [2,3]. Some species have reduced susceptibility to common antifungal drugs, creating an urgent need for new antifungal molecules focused on different fungal targets. Nature is an almost inexhaustible source of interesting new molecules that could meet this need.

Current options for antifungal therapy include polyenes, triazoles and echinocandins. However, their effectivity is reduced by several flaws, such as their relative toxicity [4]. Antimicrobial peptides (AMPs), on the other hand, possess a broad spectrum of activity against bacteria, fungi and viruses. This antimicrobial activity has not yet been altered by the development of microbial resistance [5,6].

In this review, we focus on AMPs with anti-*Candida* activity: either direct fungicidal action or indirect action. In this last case, AMPs could help to reduce the virulence of the pathogen, for example, hindering the biofilm formation capacity. Research on some AMPs has reached clinical trials, while other AMPs need further testing to lead to a better understanding of their activities [7].

To date, there are 1211 antifungal peptides registered in the Antimicrobial Peptide Database (https://aps.unmc.edu/; accessed on 15 February 2022) and 1144 in the Collection of Anti-Microbial Peptides (http://www.camp.bicnirrh.res.in/seqDb.php; accessed on 15 February 2022), from which we depict 20 from different origins (Table 1). The included peptides vary in mode of action, target, antifungal spectrum and research degree, and they are divided into four distinct groups depending on their origin: plants, humans, insects and arachnids, and a varied group of others.

The aim of this review is to highlight some of the antimicrobial peptides with activity against *Candida*. This could encourage research groups specialized in this yeast to invest in this alternative therapy and to find new and interesting molecules to cope with the important threat to our health that is *Candida* infection.

## 2. Antimicrobial Peptides from Plants

**Figure 1:**
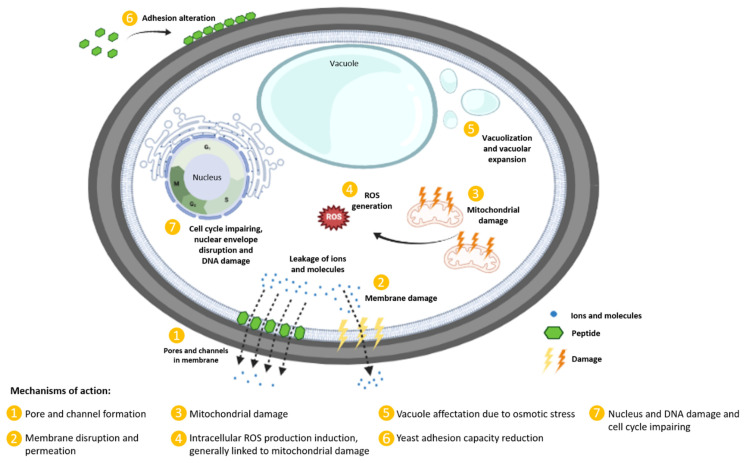
Schematic representation of the mechanisms of action of antimicrobial peptides for yeast.

Plant defensins are an important line of defense that protects plants against a possible infection. They are small, cysteine-rich peptides, the primary function of which is to avoid the microbial invasion of plant tissues. They exhibit antimicrobial activity against a wide range of pathogens, including filamentous fungi, yeasts and bacteria [8,9]. Therefore, these molecules are considered an important source of antimicrobial agents against plant and animal pathogens [10,11,12,13]. They are generally not toxic for human cells, which is the characteristic that makes these AMPs so suitable and attractive for study in the development of new drugs [12,14]. Several AMP mechanisms have been described (Figure 1). However, the exact mechanism of action of many plant defensins remains unknown.

### 2.1. HsAFP1 Peptide

*Heuchera sanguinea* antifungal peptide 1 (HsAFP1) is a 54-amino-acid-long plant defensin isolated from the seeds of *H. sanguinea*, commonly known as coral bell [10]. 

HsAFP1 can inhibit a diverse array of fungi, including species of *Candida*, such as *C. albicans* and *Candida krusei*, and of *Aspergillus*, such as *Aspergillus flavus* [12]. Several studies have investigated the mechanism of action of this AMP. Thevissen et al. [15] were among the first to elucidate that HsAFP1 permeabilizes susceptible fungal cells by interacting with its high-affinity target at the membrane. Two decades later, Cools et al. [16] showed that HsAFP1 is able to bind to several lipids at the membrane, with phosphatidic acid being its main target. Aerts et al. [17] proved the correlation between the antifungal activity of HsAFP1 and the production of intracellular reactive oxygen species (ROS) inside cells of *C. albicans*, where these free radicals are a sign of apoptosis, which leads to fungal death. In a more recent study, a transcriptomic analysis of *C. albicans* cells treated with HsAFP1 revealed changes in genes coding for proteins such as glycosylphosphatidylinositol (GPI)-anchored proteins or those involved in cation homeostasis, autophagy and the cell cycle. Likewise, in this last study, HsAFP1, in moderate doses, promoted autophagy in *S. cerevisiae* while, at higher concentrations, caused vacuole dysfunction. Moreover, HsAFP1 disrupted the cell cycle of yeast in the G2/M phase [18].

The potent antifungal activity of HsAFP1 against both *C. albicans* planktonic cells [17] and biofilm development [19,20] was assessed (Table 2). HsAFP1 caused the onset of some apoptosis markers such as ROS generation and DNA fragmentation in planktonic cells. The first piece of evidence for the activity of a plant defensin against fungal biofilms was achieved with a recombinant form of HsAFP1 in *Pichia pastoris*, and its antifungal activity was tested against both planktonic and sessile *C. albicans* cells [19]. HsAFP1 inhibited planktonic cell growth and prevented biofilm formation but did not eliminate preformed biofilms. Synergism was observed between the recombinant HsAFP1 and caspofungin, which eradicated mature biofilms [19]. Furthermore, to obtain smaller-size versions of HsAFP1 but maintain the antifungal activity, these authors selected six linear derivatives, 24 residues in length, to perform a structure–function study: HsLin01 to HsLin06. Vriens et al. [19] found that only HsLin06, corresponding to the C-terminal part of the HsAFP1, shows antibiofilm activity like that of the original AMP but, surprisingly, is not able to inhibit planktonic growth. Combination experiments with caspofungin showed a synergistic activity with HsLin01, HsLin05 and Hslin06 peptides which prevented *C. albicans* biofilm development [19]. Afterwards, 44 HsLin06 derivatives were tested to further investigate the synergistic activity between HsLin06 and caspofungin, and HsLin06_18 was the most efficacious both in vivo and in vitro [20].

### 2.2. NaD1 Peptide

NaD1 is a 47-amino-acid-long plant defensin extracted from the flowers of *Nicotiana alata*, a solanaceous plant commonly known as tobacco plant [108]. The production of this AMP is flower specific. The plant produces NaD1 mainly at the beginning of the flower development stages, with it playing an important role in protecting the organs for sexual reproduction against infections.

This peptide possesses antifungal activity against *C. albicans*, *Cryptococcus neoformans*, *Fusarium* spp. and *Aspergillus* spp. [21,22,109,110]. NaD1 penetrates the fungal cell wall and binds to its molecular target, phosphatidylinositol 4,5-bisphosphate (PIP2), a phospholipid present in eukaryotic cell membranes [111,112]. Then, the AMP enters the cytoplasm via endocytosis. Once inside, it can permeabilize the membrane and induce ROS generation, leading to oxidative stress and causing membrane breakdown and cell death [22] (Figure 1). Peptide entry is thought to initially produce a semi-permeabilization of the cell membrane, and a threshold is required to cause cell death [21,22,109]. This hypothesis regarding the two-stage cell death mechanism has also been demonstrated by a ptychography approach. This technique used quantitative phase imaging to analyze the antifungal activity of NaD1 in *S. cerevisiae* cells. The peptide caused the death of the yeast in two to five minutes after its addition [113].

### 2.3. Psd1 Peptide

Psd1 is a 46-amino-acid-long plant defensin found inside garden pea (*Pisum sativum*) seeds and was first described by Almeida et al. [11]. Later, its three-dimensional structural features were reported [114]. This peptide displays high affinity and specificity for ergosterol and glucosylceramide (GlcCer) and does not interact with cholesterol-rich membranes; thus, its toxicity towards mammalian cells is negligible [23,115]. Psd1 can enter the cell of the filamentous fungi *Neurospora crassa* and impair the cell cycle by hindering cyclin F, a vital protein for the correct progression of the cell cycle, and is believed to lead to apoptosis [116]. 

Psd1 possesses activity against several filamentous fungi, such as *Aspergillus niger*, *Aspergillus versicolor*, *Fusarium solani* and *N. crassa*, and against the pathogenic yeast *C. albicans* [11,23,116]. In fact, this peptide triggered death in *C. albicans* planktonic cells at a concentration of 20 μM in a time-dependent manner by broth microdilution method, and antibiofilm activity at a 10-times-greater concentration [24] (Table 2). This AMP provoked morphological changes on the planktonic cells of *C. albicans* which were observable using an atomic force microscope. Cells under Psd1 treatment were not able to form aggregates, highlighting its ability to compromise this important virulence factor [24]. Apart from that, Psd1 was shown to be able to inhibit two cancer cell lines in a murine model and could be a promising drug candidate for lung melanoma metastasis treatment [117].

### 2.4. RsAFP2 Peptide

The *Raphanus* *sativus* antifungal peptide 2 (RsAFP2) is a 51-amino-acid-long plant defensin found in radish seeds (*Raphanus sativus*). It is the second of the peptides isolated from this species, with RsAFP1 being the first one. RsAFP1 is the same length as RsAFP2 but differs in two amino acid residues in positions 5 and 27 of their sequences [25]. RsAFP2 has been demonstrated to possess a high and wide antifungal activity, including against both plant and human pathogens, such as *C. albicans* [14].

This AMP targets the fungal glucosylceramide at the cell surface, and it is so selective against fungal cells that the peptide is not able to bind to plant or human glucosylceramide [26]. This peptide can differentiate distinct structures from ceramides; this ability is what makes RsAFP2 a promising antifungal molecule [26]. After interacting with the fungal membrane, this peptide induces endogenous ROS generation in *C. albicans*, leading to cell apoptosis [27,28].

RsAFP2 efficiency was shown against different species of *Candida*, where the effect was higher against *C. albicans* than against *C. parapsilosis*, *C. krusei*, *C. tropicalis* or *C. dubliniensis* [12,14,26]. The effect of RsAFP2 against *C. glabrata*, which lacks glucosylceramide, was very low [29]. In addition, the in vivo efficacy of RsAFP2 against *C. albicans* was assessed using a murine candidiasis model, with RsAFP2 being as potent as fluconazole [14].

Moreover, the recombinant RsAFP2 produced in *P. pastoris* was able to prevent *C. albicans* biofilm formation [25]. Thevissen et al. [30] proved the ability of this AMP to disturb the yeast-into-hypha transition, a crucial step for the correct development of biofilms. These authors also determined the effect of the combination of RsAFP2 with amphotericin B and with caspofungin against *C. albicans* biofilm formation. A synergistic antibiofilm development activity was observed, along with a synergistic effect between RsAFP2 and caspofungin which caused biofilm eradication [30]. Recently, two mutations in positions 9 and 39 were proved to confer the AMP with more stability and a higher anti-*C. albicans* activity [31].

## 3. Antimicrobial Peptides from Humans

The human body possesses innate and adaptive immunity to face infections. Antimicrobial peptides are part of the first type of immunity and can reduce pathogen virulence by inhibiting their growth or modulating the immune system [118]. Although many of these AMPs have already been discovered and characterized, some remain undetected, and the exploration of the human peptidome could be important [119]. Five AMPs with activity against *Candida* are described below.

### 3.1. CGA-N46 Peptide

Chromogranin A (CGA)-N46 is a 46-amino-acid-long derivative from the human chromogranin A, a protein expressed in neurons. It is a recombinant peptide that corresponds to the 31st to 76th amino acid residues from the N terminus of the parent peptide chromogranin A. This peptide presented in vitro antifungal activity against *C. albicans*, *C. glabrata*, *C. parapsilosis*, *C. krusei* and *C. tropicalis*, with MICs ranging from 0.1 to 0.8 mM, *C. krusei* being the most susceptible [32]. Combination with fluconazole or terbinafine was additive. Moreover, CGA-N46 did not display hemolytic activity. On the contrary, CGA-N46 was not active against filamentous fungi, such as *Fusarium*, *Microsporum*, *Trichophyton* or *Aspergillus* [32].

This peptide exerts its antifungal activity by damaging the mitochondria, inducing vacuolization inside of the yeast cells, disturbing the nuclear envelope and inhibiting DNA synthesis by preventing DNA polymerase action [33] (Figure 1).

Treatment with CGA-N46 improved the global health of immunocompromised mice infected with *C. krusei*, increasing their average body weight and decreasing their mortality. This AMP showed an immunomodulatory effect, and it was able to alleviate the damage in different organs caused by *C. krusei* infection [34].

Li et al. [35] synthesized and tested a series of smaller by-products of this AMP by amino acid deletion to find a derivative with better antifungal activity. Four of these derivates displayed an efficient antifungal activity higher than the parent CGA-N46 peptide against *C. albicans*, *C. glabrata*, *C. parapsilosis*, *C. krusei* and *C. tropicalis*. Among them, CGA-N12 was the most potent and the least hemolytic. Recently, Okasha et al. [120] reported that a recombinant version of CGA-N46 may be useful for the treatment of human colon cancer.

### 3.2. Psoriasin Peptide

Psoriasin is a long AMP, with 101 amino acids, secreted by human keratinocytes. It is also referred to as S100A7 protein, since it belongs to the S100 protein family, a multigenic family of calcium-binding proteins [121,122]. This protein was first discovered in a patient with psoriasis [123], and it was reported that psoriatic skin presents a reduced susceptibility to skin infections [124,125]. 

This peptide has an important role in skin defense and a capacity to kill *Escherichia coli* [126,127]. Its disulfide-reduced form, redS100A7, is active against several filamentous fungi, but, surprisingly, it is not able to cause the death of the yeast *C. albicans* [128]. However, psoriasin has been shown to bind to the β-glucan present on the *C. albicans* cell wall, reducing the adhesive capacity of the yeast [36]. Therefore, despite lacking a fungicidal action against *Candida*, this peptide could be effective against biofilms, disaggregating cells and making them more accessible for antifungal drugs [36].

### 3.3. Human β-Defensins

Mammalian defensins are a group of relatively small AMPs that share structural features [129,130]. Inside their sequence, there are six cysteine amino acid residues forming three disulfide bonds [129], providing the molecules with stability by adopting a β-sheet structure [131,132]. These defensins can be sorted into α and β forms based on the mentioned cysteine residues and their disulfide bonds [130]. Both classes are present in humans [133]. Human β-defensins (HBD) are expressed mainly in epithelial tissues [133,134]. HBD-1 and HBD-2 were the first discovered, in 1995 and 1997, respectively [135]. HBD-3 was later independently described by three distinct research groups and by the means of two different methods, isolation from psoriatic scales and screening of the human genome [44,136,137], and HBD-4 was found by using bioinformatic tools [138]. 

HBD-3 is the most effective, since it can kill both Gram-negative and Gram-positive bacteria, viruses and *C. albicans* [44]. HBD-1 and 2 are also able to kill Gram-negative bacteria, but only HBD-2 possesses fungicidal activity against *C. albicans* [37,38,39,40]. However, reducing the disulfide bridges of HBD-1 transforms the peptide into a more efficient one that gains activity against *C. albicans* [139]. Despite this, the HBD-3 peptide is still the one with the strongest fungicidal effect against *C. albicans* [37,39].

The fungicidal effect of HBD-2 and HBD-3 against *C. albicans* is salt and energy dependent since sodium azide pre-treatment of yeast cells inhibits both peptides’ activity [40]. HBD-3 at 10 µM reduces 67.9% of *C. albicans* colony-forming units. However, even at lower concentrations, HBD-3 affects adherence of yeasts to plastic by upregulating the activity of the β-1,3-exoglucanase Xog1p responsible for hydrolyzing cell wall β-glucan [45]. *C. albicans* responds to HBD-2 and HBD-3, executing a response to osmotic stress by activating the high-osmolarity glycerol (HOG) pathway [41]. The quantity of HBD-2 in the presence of *C. albicans* is augmented in the lower genital tract of women [42]. In addition, the presence of HBD-2 and HBD-3 is correlated with a better protection of the gut during *C. albicans* infections, as they promote the creation of tight junctions between the epithelial cells of the intestinal mucosa [43]. The structure, gene expression and the range of the biological activities of the human β-defensins have been detailed in depth previously [133,140].

### 3.4. Histatins

Histatins are a family of histidine-rich cationic peptides secreted by our salivary glands [141]: an important part of innate immunity and crucial for antimicrobial defense in the oral cavity [46,142]. Physiological concentrations of histatins in healthy adults range between 50 and 425 mg/mL [47]. Histatin-5, a 24-amino-acid by-product of the cleavage of histatin-3, is the most prevalent [143,144] and possesses the most potent antifungal activity among all histatins [48,49].

The anti-*Candida* activity of histatins, specifically histatin-5, has been well documented in studies in vitro. In their first characterization, histatins 1, 3 and 5 were described as able to kill *C. albicans* [46]. Three years later, Xu et al. [48] reported how these three AMPs affect *C. albicans* using different ionic compositions of the culture media, concluding that histatin-5 is the most effective, in accordance with the results reported by Oppenheim et al. [46]. The antifungal activity of this histatin-5 peptide was then assayed against 26 oral isolates of *C. glabrata*, *C. parapsilosis*, *C. tropicalis*, *C. krusei* and *C. guilliermondii*; *C. tropicalis* and *C. guilliermondii* were the most susceptible species, while *C. glabrata* was the least [50]. The lower susceptibility of *C. glabrata* to histatin-5 was also reported by Helmerhorst et al. [51]. However, histatin-5 was also effective against some clinical isolates of the emergent *C. auris* [52]. 

Histatin-5 has also been proven to have antifungal activity against the sessile form of some species of *Candida* (Table 2). Pusateri et al. [53] reported that 50 μM of histatin-5 inhibited biofilm formation of *C. albicans* on acrylic dentures in vitro. Moreover, planktonic and sessile cells of *C. albicans* were susceptible to histatin-5, while planktonic cells of *C. glabrata* were unaffected, and its biofilms were less susceptible than the *C. albicans* ones [54]. Similarly, histatin-5, in the range from 25 to 800 µg/mL, was able to reduce *C. albicans* planktonic growth and reduced its adhesion to reconstructed human oral epithelial tissues [55].

Furthermore, histatin-5 protected murine oral tissue against *C. albicans* ex vivo [56]. It was also effective in reducing the fungal burden in a murine model of vulvovaginal candidiasis [57]. 

Regarding the mechanism of action of this AMP, it is known that, upon contact with the cell, histatin-5 binds to the Ssa1/2p cell wall receptor [58] and β-glucans [59] present in *C. albicans*. Afterwards, histatin-5 is internalized in an energy-dependent process by the Dur3 and Dur31 polyamine transporters [60], though the process of endocytosis may also have a role in its internalization [61,62]. Once it reaches the inner side of the cell, histatin-5 causes a flow of ions from the inside of the cell to the environment [58,62], causing an osmotic imbalance that leads to the loss of cell volume. Apart from that, the AMP also affects ATP synthesis at the mitochondria [63,64,145] and favors ROS generation [65].

Despite histatin-5’s multi-targeting mechanisms, *C. albicans* displays resistance by cleaving this peptide using several secreted aspartic proteases or Saps [146]. Modifications of histatin-5 have been analyzed, and a single amino acid residue change was enough to confer histatin-5 resistance to the proteolytic degradation by Saps of *C. albicans*. While K17R substitution improved the proteolytic resistance, K11R variation boosted the antifungal activity of histatin-5 [147]. Therefore, the combination of both changes in a single molecule, K11R-K17R, results in a peptide with both improvements. Furthermore, this variant of the peptide has also shown its ability to hinder *C. albicans* biofilms [148].

Antifungal activity of histatin-5 can also be affected by the binding of the peptide to metals [149]. Puri et al. [66] reported that histatin-5 has its candidacidal activity reduced proportionally to the iron to which it adheres. In fact, histatin-5 can adopt several conformations, and apparently, its binding to zinc in histidine residues maintains a biologically active structure. Circular dichroism analyses confirmed that histatin-5 has precise zinc-binding sites important for its correct antimicrobial activity [150].

### 3.5. LL-37 Peptide

LL-37 is a 37-amino-acid peptide with an α-helical structure, the sequence of which has two leucines at its N terminus [151]. This AMP is created after the protein cathelicidin is modified by the proteinase 3 enzyme [152] and is one of the best-described human AMPs [151,153,154,155].

LL-37 takes part in executing several defense responses such as inhibition of microbial adhesion, leukocyte chemotaxis and endotoxin counterbalancing [154,156,157]. Many types of cell synthesize LL-37: neutrophils, macrophages, mucosal epithelial cells and keratinocytes [158].

LL-37, at 2 to 5 μg/mL concentrations, is found at mucosal surfaces [159,160], and it is present in our sweat as well [161]. AMP concentration varies depending on the affected cell or tissue type and the presence of infection and/or inflammation [162,163]. LL-37 can reach a concentration of roughly 30 μg/mL in specific sites during disease [160]. In fact, the gene expression and secretion of LL-37 in human keratinocytes are enhanced when these cells are exposed to *C. albicans* cell wall phospholipomannan [68]. 

LL-37 possesses a net positive charge at neutral pH that, added to its the many hydrophobic amino acid residues and its α-helical structure, favors its binding to the negatively charged plasmatic membranes of microorganisms. As a result, this union may fracture the mentioned sheet and induce the death of the cell [151]. Several studies have corroborated the ability of LL-37 to cause the disruption of the plasmatic membrane of some bacteria and lipid vesicles by creating pores in their structure [164,165]. The peptide, additionally, also interacts with the *C. albicans* cell wall [67,69,70].

LL-37 shows a dose-dependent candidacidal effect against *C. albicans* at concentrations equal to or above 20 μg/mL, which was corroborated separately by two distinct methods: a spot assay and FUN-1 staining [69,71]. 

LL-37 causes vacuole expansion simultaneously with the membrane permeabilization in planktonic *C. albicans* cells, also causing a rapid efflux of ATP [72]. The increase in vacuole size is due to osmotic stress, derived from the leakage of cytosolic ions [59]. These results are in accordance with those reported by den Hertog et al. [73], in whose study a leakage of ATP and cytosolic ions was observed. This group also previously reported the ability of LL-37 to interact with both the cell wall and the plasmatic membrane of planktonic *C. albicans*. According to these results, LL-37 alters and even fractures the membrane morphology of the fungus, as was observed by freeze-fracture electron microscopy [67]. Apart from that, LL-37 also causes cell wall and endoplasmic reticulum stresses in *C. albicans* [74].

The ability of the peptide to affect the adhesive capacity of *Candida*, which was proved by reducing its adhesion to plastic and mouse bladders, is more remarkable than its direct candidacidal activity [70]. Yeast adhesion is compromised after the interaction of the AMP with its receptor, the β-1,3-exoglucanase Xog1p present in the cell wall, at non-lethal concentrations, while >10 µM LL-37 can kill the yeast [45,71]. Scarsini et al. [75] reported that LL-37 at 64 µM inhibited *Candida* cell adhesion to polystyrene and silicone surfaces and impeded biofilm formation but was ineffective against already formed biofilms. However, no fungicidal activity from LL-37 against planktonic *Candida* cells (with MICs above 250 μg/mL) was achieved [76]. In contrast, these authors found the AMP to be effective at inhibiting the biofilm formation of *C. albicans* and the biofilm of *Staphylococcus aureus* and *E. coli*. Moreover, LL-37 also displayed efficacy against non-mature biofilms of *C. albicans* [76] (Table 2).

The proteolytic degradation carried out by some enzymes of *C. albicans* could be the reason behind the low direct antifungal activity of LL-37 [166]. Sap2, Sap3 and Sap9 are known as the most able to carry out the digestion of LL-37. The Saps digest LL-37 into two smaller peptides, LL-25 and SK-29, which results in fungicidal activity against *C. albicans* at least equal to or somewhat more potent than the activity of the parent peptide. Despite having an antifungal activity, these two by-products are not able to survive in the media and are rapidly deteriorated [166]. In this regard, it seems that LL-37 may be converted into smaller and more efficient peptides. Murakami et al. [167] showed that LL-37, once it reaches our skin, is handled by a serine protease-dependent mechanism to yield different peptides, such as RK-31 and KS-30. These two peptides possess a better antifungal activity than that of the parent peptide against *C. albicans* [168].

Recently, it was also reported that LL-37 is able to inhibit and kill *C. auris* at concentrations ranging from 25 to 200 μg/mL [77].

Furthermore, LL-37 has been shown to exert anticancer activities [169,170]. LL-37 and some derived peptides are also capable of harming the trophozoites of *Entamoeba histolytica* [171].

## 4. Antimicrobial Peptides from Insects and Arachnids

Arthropods are the largest group of animals on the planet and the biggest source of AMPs in the animal kingdom. These peptides from arthropods commonly share a small size of about 5 kDa and an overall positive charge at neutral pH [172]. AMPs can be found both in the hemolymph, as effectors of the innate immunity, and as a component in venoms, such as lycosin-I and melittin.

### 4.1. Gomesin Peptide

Gomesin is an 18-amino-acid-long peptide isolated from the hemolymph cells of *Acanthoscurria gomesiana*, a tarantula spider found in Brazil [173]. It is a part of the immune system of the organism, and, like many AMPs, is discharged during an infection [174]. Gomesin displays homology in its sequence to peptides from other different organisms, such as the androctonin peptide from *Androctonus australis* scorpions [175], tachyplesins and polyphemusins from horseshoe crabs [176,177] and, more surprisingly, porcine protegrins [178]. Moreover, Fernandez-Rojo et al. [179] found a gomesin analogue in the Australian *Hadronyche infensa* spider.

Gomesin possesses a wide spectrum of activity against filamentous fungi and yeasts [180], as well as against Gram-positive and Gram-negative bacteria [78,173], protozoa, such as *Plasmodium* [181] and *Leishmania* [173], and even against cancer cell lines [182,183]. 

The effect of gomesin against different *C. albicans* isolates has been reported both in vitro and in vivo, alone or combined with fluconazole, and it is considered an interesting candidate for treatment of vulvovaginal candidiasis because of its low toxicity [79]. However, in vitro gomesin MICs ranged widely from 0.32 to 0.64 μM [80], 0.64 to 1.28 μM [81] and 8 to 16 μM [78]. 

Gomesin is a cationic peptide with five arginines and a lysine residue in its sequence [184] that acts by permeabilizing cell membranes with preference towards those ones with an overall negative charge [78,180,182,185]. Buri et al. [186] observed that gomesin penetrates melanoma cells and distributes within them. Gomesin impacts cell membranes by producing protrusions in the outer membrane of the lipidic layer, submitting the membrane to a great tension, which can cause the rupture of the lipid bilayer [187]. Another striking characteristic is its high serum stability that is directly associated with the presence of two disulfide bridges. At least one disulfide bridge is needed to retain antimicrobial activity, while both bridges are essential to keep serum stability [80,188]. A downside is its moderate hemolytic effect on human erythrocytes [78,80,173,188].

Since its discovery, several groups have modified gomesin to improve its activity; there are over 40 versions with a favorable outcome. The peptide has been cyclized, improving its stability [78,189] and therapeutical capacities, including its anti-*C. albicans* activity [78]. To find an analogue without the hemolytic adverse effect, gomesin was modified by making amino acid substitutions in its sequence, and two linear derivatives were synthesized, displaying less hemolytic activity and keeping the antifungal effect [188].

### 4.2. Heliomicin

Heliomicin is a cysteine-rich, 44-amino-acid peptide isolated from the hemolymph of the larvae of the lepidopteran *Heliothis virescens* [82] that has shown a remarkable in vitro antifungal activity against filamentous fungi, including *Fusarium culmorum* and *N. crassa*. This peptide inhibited *C. albicans* growth at 2.5 and 5 µM but did not inhibit *C. glabrata* even at the highest concentration used (50 µM). However, heliomicin did not show antimicrobial activity against bacteria [190].

Approximately 50% of the sequence of heliomicin is coincidental with the sequence of drosomycin, an antifungal peptide from *Drosophila melanogaster* [191], with which it shares a global charge of +1 [190]. Heliomicin is also very similar to the RsAFP2 peptide, the plant defensin from *R. sativus* [26].

Heliomicin has been the subject of several modifications in the search for analogues with improved antimicrobial activity [83,190]. Of the 15 analogues obtained, the molecule named Hel-LL is the most interesting one [190]. The switches of the lysine and the arginine in positions 23 and 24 by two leucines made Hel-LL gain activity against Gram-positive bacteria, such as *Micrococcus luteus*, while maintaining almost the same antifungal activity as the original heliomicin. Later, the antimicrobial activities and 3D structures of the analogue ARD1 peptide found in *Archeoprepona demophoon*, which differs in only two amino acids from heliomicin, and its derivatives ETD135 and ETD151, were characterized [83]. These three molecules have the same antimicrobial spectrum as heliomicin but higher antifungal activity. The growth of two *C. albicans* isolates was inhibited by 50% by heliomicin at 12.5 µg/mL. Moreover, *C. albicans* was at least twice as susceptible to ARD1 as to heliomicin and 4–8 times more susceptible to ETD135 and ETD151. Landon et al. [83] hypothesized that these AMPs interact with the yeast membrane, and Thevissen et al. [26] reported the interaction of heliomicin with fungal glucosylceramide. 

In fact, some heliomicin analogues have undergone clinical trials [192]. The in vitro antifungal activity of several analogues of heliomicin against *C. albicans*, *C. parapsilosis*, *C. krusei* and *C. tropicalis* was assessed, with MICs ranging from 0.16 to 0.64 µM. The derivate ETD151 was shown to be non-toxic and more effective than amphotericin B and azoles, such as fluconazole and itraconazole, against *C. albicans* candidiasis in an in vivo murine model [192]. A proteomic analysis of the activity of ETD151 in *Botrytis cinerea* revealed that this analogue affects six different pathways but does not directly affect the respiratory chain [193].

### 4.3. Jelleine Peptides

Jelleines are a family of four AMPs, eight or nine amino acids in length, found in the royal jelly of *Apis mellifera* honeybees [84]. These AMPs are derived from the major royal jelly protein 1 precursor [194]. As royal jelly is a compound secreted to the exterior by the worker bees, and is the main nourishment of the honeybee queen, it plays a role in protecting against external aggression by pathogens. These AMPs, while only differing in one amino acid, are very distinct in terms of antimicrobial activity [84]. Most of their residues are hydrophobic, and they do not present sequence similarities with other known AMPs from honeybees, such as apidaecins and royalisin [84,195]. 

Fontana et al. [84] tested these four peptides against 11 species of bacteria, including *S. aureus*, *E. coli* and *Pseudomonas* *aeruginosa*, and against *C. albicans*; jelleine-I and -II were the most active ones, while jelleine-III had a more limited spectrum, and it was not effective against *C. albicans*. Jelleine-IV displayed no activity against the examined microorganisms. Moreover, all four peptides presented a low hemolytic activity against rat erythrocytes [84].

Jelleine-I has been studied in a more extensive way than the others have. Cabrera et al. [194] reported that jelleine-I can form pores in the cell membrane, lysing bacteria and yeasts. The molecular dynamics simulations indicated the importance of the Pro1 residue of jelleine-I, highlighting it as the decisive factor for the antimicrobial activity. 

Jelleine-I was analyzed against *Candida* species to detail its antifungal activity and mode of action. Jia et al. [85] suggested that jelleine-I increases cellular ROS production, also affecting the mitochondria (Figure 1). The target AMP showed remarkable antifungal activity against *C. albicans*, *C. glabrata*, *C. parapsilosis*, *C. tropicalis* and *C. krusei* both in vivo in a murine model of candidiasis and in vitro with a microdilution method, as well as a very low hemolytic activity (Table 2). 

Jia et al. [196] designed and synthesized jelleine-I halogenated derivative molecules, with some of these showing better antimicrobial activity than the original peptide while improving its stability and maintaining the same cytotoxicity. Moreover, two jelleine-I analogues were designed by changing the phenylalanine in the second position by a tryptophan, firstly, and the lysine in the third and the histidine in the seventh positions by arginines in addition to the aforementioned amino acid switch, secondly [86], creating two synthetic peptides: JIF2W and JIF2WR. While JIF2W has lower antimicrobial activity but increased anticancer activity at the expense of a higher toxicity towards human cells, JIF2WR possesses similar antimicrobial activity to that of jelleine-I but with the advantage of a lower propensity to aggregation. Apart from that, another jelleine-I analogue showed great antimicrobial activity against multi-resistant *P. aeruginosa* while maintaining the slight toxicity of the natural peptide [197].

### 4.4. Lasioglossin Peptides

Lasioglossins [LL] are a family of antimicrobial peptides discovered by Cerovský et al. [198] in the venom of *Lasioglossum laticeps*, a eusocial bee that lives in Europe. There are three different peptides with 15 amino acids each: LL-I, LL-II and LL-III. These peptides differ slightly in their sequences. A bigger discrepancy is found between the sequences of LL-I and LL-III because of their sixth and fourteenth amino acid residues. Moreover, the sequences of LL peptides are not homologous to other AMPs collected in the Antimicrobial Peptide Database [198].

These three peptides showed antimicrobial activity in low micromolar concentrations against four tested bacteria, *Bacillus subtilis*, *S. aureus*, *E. coli* and *P. aeruginosa*, while having low hemolytic activity that was indistinguishable in concentrations lower than 200 μM [198]. The low hemolytic activity is in accordance with the results reported by Slaninová et al. [199].

However, among the three LLs, LL-III presented the most remarkable antimicrobial activity in vitro against the above-mentioned four species of bacteria [198] and yeasts, such as *C. albicans*, *C. dubliniensis*, *C. glabrata*, *C. krusei*, *C. tropicalis* and *Saccharomyces* spp. [87,88], as well as against cancer cells [198,199].

LL-III permeabilized the cell membranes of the microorganisms, which included *S. cerevisiae*, and some species of *Candida*, such as *C. albicans*, *C. glabrata* and *C. krusei*, among others, in a dose-dependent manner, as observed by means of fluorescence screening assays [88]. The membrane permeabilization was later confirmed by another group [200]. Later, Vrablikova et al. [89] referenced the ability of LL-III to inhibit *C. albicans* switching from yeast form to hypha, which is considered important for the formation of fungal biofilm, an important virulence factor. Moreover, Vrablikova et al. [89] observed that topical LL-III was enough to reduce the yeast cell burden in a murine model of vulvovaginal candidiasis, shortening the duration of disease. Additionally, LL-II was able to bind in vitro to DNA, which could be related to its anticancer activity [201].

More recently, Vaňková et al. [202] tested the effect of a synthetic derivative of LL-III, LL-III/43, against *C. albicans*. This synthetic AMP was able to hinder the biofilm formation of the yeast at a concentration of 50 μM. Moreover, 25 μM of LL-III/43 in combination with 50 μM of clotrimazole attenuated the production of phospholipases and proteases and completely restrained the hemolytic activity. Additionally, the combination of 25 μM of LL-III/43 and 3.1 μM of clotrimazole prevented biofilm formation in urinary catheters [202]. This, once again, highlights the importance of molecule combination procedures as a good alternative against resistant microorganisms.

### 4.5. Lycosin-I Peptide

Lycosin-I is a 23-amino-acid-long antimicrobial peptide, isolated from the venom of the *Lycosa singoriensis* spider, mainly known for its ability to weaken cancer cells [203]. Its strong anticancer activity points to lycosin-I as a possible structural base for the development of future antitumor drugs. It was able to contain tumors in vitro and in vivo, both activating cell apoptosis and stopping cell multiplication [203]. Only 5 μM of this peptide was successful in inhibiting the metastasis capacity of the PC-3 and DU-145 cancer cell lines [204]. In addition, lycosin-I has been the subject of modifications useful for enhancing its anticancer activity. Zhang et al. [205] synthesized an arginine-modified lycosin-I, which they named R-lycosin-I, by replacing its seven lysine residues in positions 1, 5, 8, 12, 16, 18 and 20 with arginines to try to improve its entry into cancer cells. 

The activity of lycosin-I was investigated against a total of 27 reference strains of bacteria and fungi, and the effect was compared to that of magainin-2, an antimicrobial peptide derived from *Xenopus* frogs [206]. Overall, lycosin-I was able to inhibit the growth of most microorganisms at low micromolar concentrations and in a short lapse of time, proving to be more potent than magainin-2. In addition, lycosin-I was synergistic with traditional antibiotics [206].

Tan et al. [90] studied the effect of lycosin-I against 66 isolates of *C. albicans*, *C. glabrata*, *C. krusei*, *C. parapsilosis* and *C. tropicalis*. The peptide exhibited the highest antifungal activity against both fluconazole-resistant and fluconazole-susceptible strains of *C. tropicalis*, with MICs ranging from 8 to 128 μg/mL, while *C. glabrata* was the most resistant with MICs above 512 μg/mL. In this study, and similarly to the results reported by Tan et al. [206], lycosin-I was able to exert its antifungal activity more quickly than fluconazole. As no difference in the killing of fluconazole-susceptible and fluconazole-resistant strains was found, the authors proposed that the AMP could kill *Candida* by a different mechanism to that of fluconazole, by firstly binding to the anionic membrane of the microorganism and then entering the cytoplasm. This peptide also showed an effect against the biofilms of *C. tropicalis*, the most susceptible of the *Candida* species tested. Lycosin-I induced general morphological changes in treated biofilms where the hypha ramifications were less numerous and complex than those in untreated biofilms. Aside from these changes, lycosin-I was able to reduce biofilm formation too [90].

In addition, lycosin-I is also effective against *Toxoplasma gondii* [207]. Three distinct isomers of lycosin-I, (L-, D- and the turned around S-lycosin-I) resulted in a higher efficacy than that of several conventional drugs against multi-drug-resistant *Acinetobacter baumanii* [208]. Furthermore, the 21-amino-acid-long lycosin-II, a peptide related to lycosin-I, which comes from the same origin, was also tested against *A. baumanii* and another eight bacteria, including *S. aureus*, *P. aeruginosa* and *E. coli*, displaying a strong and quick antibacterial activity [209].

### 4.6. MAF-1A Peptide

MAF-1A is a 26-amino-acid-long linear antimicrobial peptide derived from the MAF-1 protein present in the *Musca domestica* housefly, corresponding to amino acids 128–153 of that protein [210].

This peptide has shown a noteworthy antifungal effect [91,92,93,210]. MAF-1A induced a series of complex transcriptional responses to inhibit *C. albicans* growth, including the downregulation of ergosterol synthesis and ROS production [91]. Some transcriptional responses to MAF-1A were also studied in *C. parapsilosis*, showing that the membrane and some organelles, such as the mitochondria, were affected when the yeast was treated with this AMP [92]. Furthermore, MAF-1A also compromises the cell wall and binds to *C. albicans* nucleic acids [93]. Apart from that, MAF-1A was able to inhibit *Rhizopus stolonifer*, *Sclerotina sclerotiorum*, *Sclerotium rolfsii* and *Thanatephorus cucumeris* and displayed a good antiviral activity against the H1N1 influenza virus [210].

### 4.7. Melectin Peptide

The antimicrobial peptide melectin, an 18-amino-acid-residue-long antimicrobial peptide, is the major component of *Melecta albifrons* spring bee venom. It is mainly composed of hydrophobic and basic amino acids. The proline residue in position 11 of its sequence introduces a kink in its α-helical structure [211]. Melectin shares this structural uniqueness with the antapin peptide from the *Anthophora plumipes* bee [212]. Both peptides present amphipathic α-helical structures which are essential for their lytic activity [211,212]. 

Melectin showed antimicrobial activity against both Gram-positive and Gram-negative bacteria [211] and against *C. albicans* [87]. The AMP could inhibit the growth of this yeast with a concentration as low as 8.3 ± 1.8 μM, and, as in the case of most hymenopteran venom AMPs, it could kill *C. albicans* in minutes [87]. 

Melectin shows low hemolytic activity [211], and, in mammalian cancer cells, this peptide mainly targets the cell membrane, and, after penetrating the cell, it is able to compromise the whole cell’s integrity [199]. Asides from that, melectin is also able to bind to plasmid DNA [213]. 

### 4.8. Melittin Peptide

Melittin, with its 26 amino acids, is the major constituent of *Apis mellifera* European bee venom [214]. It is currently one of the most studied AMPs and, among all its bioactive activities, presents a marked antifungal activity. Do et al. [94] studied the effect of melittin, along with other AMPs (cecropin A, histatin-5 and protegrin-1), on several *C. albicans* strains and two skin cancer cell lines. Melittin displayed the best antifungal effect (MIC 0.4 μM), even besting amphotericin B (MIC 1.7–2.2 μM).

Melittin is a peptide with a high cytolytic capacity that induced apoptosis in *C. albicans* through the activation of several mechanisms (Figure 1). It is a pore-forming peptide [215], which, within nanomolar concentration range, forms cell membrane pores, leading to the leakage of ions [216]. Moreover, at micromolar concentrations, pores are bigger, enabling larger molecules to leak from the cell [216,217]. Apart from this, once it reaches intracellular molecules, it binds to them while triggering endogenous ROS generation [95]. This production helps to reduce the membrane potential in the mitochondria, confirming its involvement in the induction of apoptosis in *C. albicans* [96]. Then, Ca^2+^ ions released from the endoplasmic reticulum are relocated into the mitochondria, damaging the organelle even more. The release of cytochrome c from the mitochondria in the cytosol provokes the activation of caspase protease, which is essential in the apoptosis process. Due to its recognized mode of action and potency, melittin is commonly used as a positive control in research with AMPs [96]. The specific activity of this AMP against several fungi has been reported [97].

## 5. Antimicrobial Peptides from Other Sources

Despite this review having put its focus on three main groups, AMPs can be found in almost all living forms in nature. Several of them present antifungal activity against *Candida* and could prove useful in the search for new treatments for candidiasis.

### 5.1. Bovine Cateslytin

Catestatin is the peptide created by the cleaving of amino acids 344 to 364 of chromogranin A, and it is the precursor of cateslytin [218]. Chromogranin A [219] is the most representative in the granin family, a group of acidic proteins secreted by several neuroendocrine and immune cells upon different stimuli in vertebrates [220]. In humans, the chromogranin A gene is located inside the 14th chromosome [221]. Chromogranin A is composed of 431 amino acids and has several cleavage sites in its polypeptide chain, thus, leading to the birth of several smaller peptides with different biological activities [220,222]. 

Bovine cateslytin is a 15-amino-acid peptide corresponding to the N-terminus of catestatin [98]. Both catestatin and cateslytin have been shown to possess antimicrobial properties [99,223], along with their other main functions as bioactive peptides derived from chromogranin A, that affect the immune, endocrine and cardiovascular systems [224]. Cateslytin lacks a defined structure while in solution and, in the presence of negatively charged membranes, adopts an antiparallel β-sheet structure to aggregate upon the mentioned membranes [225]. Arginine residues of the peptide play an important role in the binding to negatively charged lipids on the membrane of the microorganisms due to electrostatic forces. Cateslytin can form channels in the cell membrane [226]. Bovine cateslytin and its human counterpart were able to exhibit their antimicrobial activity against bacteria, including *M. luteus*, *Bacillus megaterium* and *E. coli*, several filamentous fungi, such as *Aspergillus fumigatus* or *Fusarium culmorum*, and yeasts, including *C. albicans*, *C. glabrata* and *C. tropicalis*, at low micromolar concentrations. The bovine peptide proved to be more effective than the human one [99]. In this study, 8, 30 and 10 µM of the AMP were sufficient to completely inhibit the growth of the aforementioned *Candida* species, respectively. Cateslytin, since it displays a great selectivity towards fungal cell membranes containing ergosterol [227], is unable to affect mammalian cell membranes, and, therefore, no hemolytic activity has been reported for this peptide [99]. 

The combination of cateslytin with other antimicrobial drugs, such as minocycline against *S. aureus* and voriconazole against *C. albicans* and *C. tropicalis*, was also tested [228]. Fractional inhibitory concentration values of 0.25 and 0.5 were obtained for the mentioned microorganisms, respectively, confirming a synergistic effect between voriconazole and cateslytin.

To improve the effectivity of the peptide, Zaet et al. [229] modified the molecule by replacing all its levogyre amino acid residues with their dextrogyre forms, naming the new peptide D-cateslytin. Aside from retaining the antibacterial activity, it also resisted the effect of the proteases secreted by the seven tested bacteria, including *E. coli*, *S. aureus*, *Fusobacterium nucleatum*, *Prevotella intermedia* and *Parvimonas micra*. Later, Dartevelle et al. [98] tested the antifungal capacity of D-cateslytin in vitro against planktonic cells of *C. albicans* and compared it to the effectivity of the natural form, L-cateslytin. Both peptides were efficient against *Candida*, with MIC values of 5.5 μg/mL for D-cateslytin and 7.9 μg/mL for L-cateslytin, thus, proving the modified peptide was more effective than the natural one. D-cateslytin endured the effect of the secreted proteases in the supernatant of *C. albicans*, while L-cateslytin did not, in accordance with the results obtained in the previously mentioned study [229] where the D isomer was more resistant to the secreted proteases than the L isomer. This D isomer of cateslytin, which is stable in human saliva, unlike the L isomer, was found to present an additive effect against *C. albicans* when combined with voriconazole, since the FIC value obtained with the mixture of both antifungal agents was 0.75 [98]. Recently, the D isomer of bovine cateslytin was reported to be active against *C. albicans*, *C. tropicalis* and *C. glabrata* and was suggested as a possible template for the development of novel treatments for oral candidiasis [100].

### 5.2. Dermaseptin Peptides

Amphibian skin contains a wide array of gene-encoded antimicrobial peptides with different pharmacological properties [230,231]. Along with the dermaseptins, there are several antimicrobial peptide families that shape the chemical defense system of amphibians, such as the bombinins from the *Bombina* toad species [232], magainins from *Xenopus laevis* [233] or brevinins and esculentin from the European frog *Rana esculenta* [234]. The dermaseptins are an AMP family found in the skin of *Phyllomedusinae* frogs, commonly known as leaf frogs, and its secretions. These peptides are produced and stored in the granular glands of these organisms and released when required under a certain stress inducement, such as an infection, or when required to prevent it [235]. To date, over 50 different dermaseptin peptides between 27 and 34 amino acid residues in length [104] have been described [236]. They are cationic molecules with 3–6 lysine residues and a conserved tryptophan amino acid in the third position starting from the N terminus of the molecule [101]. They all tend to form α-helical structures in non-polar media [5]. Dermaseptins are an important part of the defense mechanisms of these amphibians against different pathogens, such as bacteria, viruses, fungi and protozoa, and show little to no hemolytic activity [102,237], making this family of peptides an even more interesting asset to study in the search for promising new antimicrobial agents. 

Regarding the mechanism of action, it seems that these peptides attach to acidic components of membranes, compromising the osmotic balance of the cell by acting above the plasmatic membrane in a carpet-like manner [230]. Dermaseptins disarray cell membranes, leading to microorganism death [238]. A recent study with dermaseptin-S4 against *C. albicans* revealed its ability to inhibit yeast-to-hypha transition and hinder the biofilm formation [239].

Specifically, dermaseptins-S1, -S2, -S3, -S4 and -S5 (DS1, DS2, DS3, DS4 and DS5, respectively) showed a lytic action against some Gram-positive and Gram-negative bacteria, filamentous fungi and yeasts in vitro at relatively low micromolar concentrations. The concentrations differed depending on the peptide, with *C. albicans* being inhibited at MICs of 5 to 20 μM [102].

DS1, having the lowest hemolytic activity among all the dermaseptins, could be the most suitable to be investigated for clinical usefulness [101]. This peptide, at 50 μg/mL and 100 μg/mL, decreased *C. albicans* proliferation by over 80%, even though this growth inhibition was less than that obtained with amphotericin B [103]. In addition to decreasing the fungal burden, DS1 inhibited the transition from blastospore to hyphal form, reducing the ability of the yeast to form biofilms (Table 2). Savoia et al. [101] proved that the minimum bioactive sequence of this peptide is DS1(1-15)-NH_2_ and that the tryptophan residue in the third position is important for the activity of the peptide.

Shi et al. [104] found two new members of the family in the skin secretion of the *Pachymedusa dacnicolor* frog, which were named dermaseptin-PD-1 and dermaseptin-PD-2. These authors tested synthetic derivatives of both peptides for antimicrobial activity, concluding that they displayed moderate-to-high inhibition of the studied standard-model microorganisms, which included a *S. aureus*, *E. coli*, *P. aeruginosa* and *C.* *albicans*, among others, and cancer cell lines while showing low hemolytic activity. In this study, concentrations of 39.2 μM for dermaseptin-PD-1 and 10.1 μM for dermaseptin-PD-2 inhibited *C. albicans* growth.

### 5.3. NFAP Peptides

NFAP and NFAP2 are two peptides, 57 and 52 amino acids long, with a molecular mass of 6.6 and 5.6 kDa, respectively, secreted by the filamentous fungi *Neosartorya fischeri* [105,240]. The cysteine residues included inside their sequences are of remarkable importance since they form disulfide bonds within the molecule, endowing the peptides with a significant stability, especially at high temperatures. 

Both AMPs share a main protective function but differ in their target. While NFAP possesses activity against several filamentous fungi, including some *Aspergillus* species, and is ineffective against yeasts, NFAP2 has proved to be its inverse [241]. Another important difference between both antifungal proteins is the conditions in which they are secreted; NFAP was found when *N. fischeri* was grown in an antifungal protein induction medium [240], while NFAP2 was purified from fungi cultivated in a minimal medium [105].

NFAP2 inflicted damage enough to kill clinically relevant *Candida* species, such as *C. albicans*, *C. parapsilosis* and *C. glabrata*, in low cationic broth (LCM) medium, with MICs ranging from 0.391 to 1.563 μg/mL. Moreover, the peptide was unable to induce apoptosis, suggesting the compromising of the membrane as the main antifungal mode of action [105]. Tóth et al. [106] also tested the in vitro activity against *Candida* of both recombinant and synthetic NFAP2. The results differed from the ones Tóth et al. [105] obtained, mainly because of the use of a different growth medium. In comparison, MICs were higher in RPMI 1640 medium [106] than in LCM medium [105].

Moreover, Tóth et al. [106] proved that the combination of the recombinant NFAP2 yielded by the *Penicillium chrysogenum* system and fluconazole resulted in a synergistic relation with FICI values of 0.28 and 0.19 for *C. albicans* and *C. parapsilosis*, respectively. Therefore, fluconazole combination therapy could be useful for overcoming the problem of relatively high MICs, which occur when using the peptide on its own. On the contrary, the FICI value of the combination in the case of *C. krusei* was 0.52, showing that the relation between these agents is indifferent. Additionally, this synergy and the in vivo applicability was also proved in a murine vulvovaginitis model where, additionally, the application of the peptide did not exert any cytotoxic effect, highlighting its possible use as a topical drug [107].

## 6. Conclusions

The alarming prevalence of antimicrobial resistance in different clinical niches should encourage efforts to develop innovative and efficient mitigation strategies. Concretely, the incidence of emerging multi-drug-resistant *Candida* species keeps increasing, so the need to search for novel antifungal drugs that target these pathogens in different approaches is now more urgent than ever. The natural world is an almost endless source of interesting new candidate molecules that could fulfil this need. In this review, we described 20 AMPs and their modifications obtained from different origins that have shown antifungal activity against different *Candida* species. As the research on this kind of molecule increases with the years, some AMPs have reached clinical trials, while others need more detailed and effective studies for a better understanding of their antimicrobial activity. These AMPs present some handicaps, such as their low production yield and still-inefficient pharmaceutical presentation, which avoids susceptibility to proteolytic enzymes. A number of research teams are working on different nanobiotechnological approaches, such as the inclusion of AMPs inside a lipid carrier or the use of peptoids, in order to mitigate the drawbacks associated with proteolytic degradation or possible AMP toxicity and to enhance the pharmacodynamics and stability of the AMP [242,243]. However, AMPs possess many advantages over conventional antifungal drugs, particularly their relative safety. In-depth studies on AMPs, including those presented in this review and those expected in the future, could overcome the aforementioned challenges. They could offer hope for the treatment of infectious diseases, such as superficial and deep mycoses, which are serious and increasing menaces for humankind.

## Figures and Tables

**Table 1:** Antimicrobial peptides with potential activity against *Candida*.

Origin of Peptide		Name of Peptide	Sequence of Peptide	Number of aa
Plants	*Heuchera sanguinea* (coral bell)	HsAFP1	DGVKLCDVPSGTWSGHCGSSSKCSQQCKDREHFAYGGACHYQFPSVKCFCKRQC	54
	*Nicotiana alata* flowers (tobacco plant)	NaD1	RECKTESNTFPGICITKPPCRKACISEKFTDGHCSKILRRCLCTKPC	47
	*Pisum sativum* seeds (pea)	Psd1	KTCEHLADTYRGVCFTNASCDDHCKNKAHLISGTCHNWKCFCTQNC	46
	*Raphanus sativus*	RsAFP2	QKLCQRPSGTWSGVCGNNNACKNQCIRLEKARHGSCNYVFPAHKCICYFPC	51
Human	*Homo sapiens*	CGA-N46	PMPVSQECFETLRGHERILSILRHQNLLKELQDLALQGAKERAHQQ	46
		Psoriasin	MSNTQAERSIIGMIDMFHKYTRRDDKIEKPSLLTMMKENFPNFLSACDKKGTNYLADVFEKKDKNEDKKIDFSEFLSLLGDIATDYHKQSHGAAPCSGGSQ	101
		β-Defensin-1	DHYNCVSSGGQCLYSACPIFTKIQGTCYRGKAKCCK	36
		β-Defensin-2	GIGDPVTCLKSGAICHPVFCPRRYKQIGTCGLPGTKCCKKP	39
		β-Defensin-3	GIINTLQKYYCRVRGGRCAVLSCLPKEEQIGKCSTRGRKCCRRKK	45
		β-Defensin-4	EFELDRICGYGTARCRKKCRSQEYRIGRCPNTYACCLRKWDESLLNRTKP	50
		Histatin-5	DSHAKRHHGYKRKFHEKHHSHRGY	24
		LL-37	LLGDFFRKSKEKIGKEFKRIVQRIKDFLRNLVPRTES	37
Insects and arachnids	*Acanthoscurria gomesiana* (spider)	Gomesin	ZCRRLCYKQRCVTYCRGR	18
	*Heliothis virescens* (lepidopteran)	Heliomicin	DKLIGSCVWGAVNYTSDCNGECKRRGYKGGHCGSFANVNCWCET	44
	Royal Jelly of *Apis mellifera* (honeybee)	Jelleine-I	PFKISIHL	8
		Jelleine-II	TPFKISIHL	9
		Jelleine-III	EPFKISIHL	9
		Jelleine-IV	TPFKISIH	8
	*Lasioglossum laticeps* venom (bee)	Lasioglossin I	VNWKKVLGKIIKVAK	15
		Lasioglossin II	VNWKKILGKIIKVAK	15
		Lasioglossin III	VNWKKILGKIIKVVK	15
	*Lycosa singoriensis* venom (spider)	Lycosin-I	KGWFKAMKSIAKFIAKEKLKEHL	23
	*Musca domestica* (housefly)	MAF-1A	KKFKETADKLIESAKQQLESLAKEMK	26
Insects and arachnids	*Melecta albifrons* venom (bee)	Melectin	GFLSILKKVLPKVMAHMK	18
		Melittin	GIGAVLKVLTTGLPALISWIKRKRQQ	26
Bovine		Bovine cateslytin	RSMRLSFRARGYGFR	15
Amphibian skin	*Phyllomedusinae* frogs (leaf frogs)	Dermaseptin DS-1	ALWKTMLKKLGTMALHAGKAALGAAADTISQGTQ	34
		Dermaseptin PD-1	GMWSKIKETAMAAAKEAAKAAGKTISDMIKQ	33
		Dermaseptin PD-2	GMWSKIKNAGKAAAKAAAKAAGKAALDAVSEAI	33
Filamentous fungi	*Neosartorya fischeri*	NFAP2	IATSPYYACNCPNNCKHKKGSGCKYHSGPSDKSKVISGKCEWQGGQLNCIAT	52

**Table 2:** Effectivity of the peptides against planktonic and/or sessile cells of *Candida* species.

Origin of Peptide	Name of Peptide	Sensitive *Candida* Species	Target Cell Type	MIC Range (µM) *	References
Plants	HsAFP1	*C. albicans*, *C. krusei*	Planktonic and sessile	10	[12,17,19,20]
	NaD1	*C. albicans*	Planktonic	2	[21,22]
	Psd1	*C. albicans*	Planktonic and sessile	10–20	[23,24]
	RsAFP2	*C. albicans*, *C. tropicalis*, *C. parapsilosis*, *C. krusei*, *C. dubliniensis*	Planktonic and sessile	5–10	[12,14,25,26,27,28,29,30,31]
Human	CGA-N46	*C. albicans*, *C. glabrata*, *C. parapsilosis*, *C. krusei*, *C. tropicalis*	Planktonic	100–800	[32,33,34,35]
	Psoriasin	*C. albicans*	Sessile	-	[36]
	β-Defensin-2	*C. albicans*	Planktonic	0.9–13.8	[37,38,39,40,41,42,43]
	β-Defensin-3	*C. albicans*	Planktonic and sessile	0.3–6.6	[37,39,40,41,43,44,45]
Human	Histatin-5	*C. albicans*, *C. auris*, *C. parapsilosis*, *C. krusei*, *C. tropicalis*, *C. guilliermondii*	Planktonic and sessile	1.6–50	[46,47,48,49,50,51,52,53,54,55,56,57,58,59,60,61,62,63,64,65,66,67]
	LL-37	*C. albicans*, *C. auris*	Planktonic and sessile	0.8–100	[45,67,68,69,70,71,72,73,74,75,76,77]
Insects and arachnids	Gomesin	*C. albicans*	Planktonic	0.32–16	[78,79,80,81]
	Heliomicin	*C. albicans*	Planktonic	2.5- > 50	[82,83]
	Jelleine-I	*C. albicans*, *C. glabrata*, *C. parapsilosis*, *C. tropicalis*, *C. krusei*,	Planktonic	2.5–64	[84,85,86]
	Jelleine-II	*C. albicans*	Planktonic	2.5	[84]
	Lasioglossin III	*C. albicans*, *C. glabrata*, *C. krusei*, *C. tropicalis*, *C. dubliniensis*	Planktonic and sessile	0.2–11.5	[87,88,89]
	Lycosin-I	*C. albicans*, *C. parapsilosis*, *C. krusei*, *C. tropicalis*	Planktonic and sessile	8–256	[90]
	MAF-1A	*C. albicans*	Planktonic	0.18–35	[91,92,93]
	Melectin	*C. albicans*	Planktonic	6.5–10.1	[87]
	Melittin	*C. albicans*	Planktonic	0.4–3.5	[94,95,96,97]
Bovine	Bovine cateslytin	*C. albicans*, *C. glabrata*, *C. tropicalis*	Planktonic	1.2–8	[98,99,100]
Amphibian skin	Dermaseptin DS-1	*C. albicans*	Planktonic and sessile	10- > 24	[101,102,103]
	Dermaseptin PD-1 and PD-2	*C. albicans*	Planktonic	39.2–10.1	[104]
Filamentous fungi	NFAP2	*C. albicans*, *C. glabrata* *C. parapsilosis*,	Planktonic and sessile	0.07–144	[105,106,107]

* Range of minimum inhibitory concentration (MIC) of each peptide against *Candida* spp.

## Data Availability

Not applicable.
